# Portopulmonary hypertension in cirrhotic patients: Prevalence, clinical features and risk factors

**DOI:** 10.3892/etm.2013.918

**Published:** 2013-01-22

**Authors:** HUI-SONG CHEN, SU-RONG XING, WEI-GUO XU, FAN YANG, XIAO-LONG QI, LE-MIN WANG, CHANG-QING YANG

**Affiliations:** Division of Gastroenterology and Digestive Diseases Institute, Tongji Hospital of Tongji University School of Medicine, Shanghai 200065, P.R. China

**Keywords:** liver cirrhosis, portopulmonary hypertension, risk factors, hemoglobin

## Abstract

The incidence and clinical features of portopulmonary hypertension (POPH) have not been adequately described and it is currently unknown whether an association exists between the severity of POPH and liver function. Additionally, POPH risk factors are yet to be identified. The aim of this study was to determine the prevalence, describe the clinical features and investigate the potential risk factors of POPH. We conducted a study of 100 cirrhotic patients hospitalized between March 2011 and May 2012 at Tongji Hospital in Shanghai. The clinical characteristics of patients with and without POPH were analyzed. Clinical variables with a possible association with POPH were measured and pulmonary artery systolic pressure (PASP) was determined by cardiac Doppler echocardiography. Of the 100 patients enrolled in this study, 10 were diagnosed with POPH. Seven of the cases were mild, two were moderate and only one was severe; eight were attributed to viral infections. POPH was not detected in patients with schistosomal or alcoholic cirrhosis. Hemoglobin (Hb) levels were lower in patients with POPH compared to those without POPH (P<0.01) and the severity of POPH was not significantly correlated with Child-Pugh grade (R=−0.06, P=0.09). Hb levels, incidence of hepatitis C virus (HCV) infection and portal vein thrombosis differed between the two groups (P<0.05). Hb levels were identified as an independent risk factor associated with POPH and portal vein thrombosis may play an important role during the development of POPH. However, the severity of POPH was not associated with liver function.

## Introduction

Portopulmonary hypertension (POPH) refers to the condition of simultaneous pulmonary arterial and portal hypertension. Hemodynamically, it is defined as a mean pulmonary artery pressure (MPAP) >25 mmHg and normal volume status or pulmonary capillary wedge pressure (PCWP) <15 mmHg in patients with chronic liver disease and portal hypertension (1–3). POPH has been reported in ∼6–9% of patients with advanced liver disease, that are referred for liver transplants (4,5). This variation in prevalence results from the different groups of cirrhotic patients studied and different diagnostic procedures used.

Clinical manifestations of portal hypertension typically precede those of pulmonary artery hypertension by 2–15 years. The most common symptoms of POPH include dyspnea upon exertion, syncope, chest pain, fatigue, hemoptysis and orthopnea. The presence of molecular markers is also likely, as one study supported the hypothesis that pulmonary vasculature may be exposed to either cytokines or excess circulating vasoconstrictors, including interleukin-6 and endotoxin produced by the diseased liver (6). However, to date, the predictors of and biological mechanism responsible for the development of this complication remain unknown.

There are no known clinical factors that determine the risk of POPH in patients with advanced liver disease. Similarly, the mechanism for pulmonary vascular obliteration in patients with portal hypertension, characterized by systemic vasodilation remains unclear. It follows that the identification of patient characteristics associated with an increase or decrease in the probability of developing POPH may not only be clinically useful, but may also shed light on the etiology of this relatively common comorbidity of portal hypertension. Therefore, the aims of this study were to explore the clinical features of POPH and to identify risk factors associated with POPH in cirrhotic patients.

## Patients and methods

### Patients

From March 2011 to May 2012, 145 consecutive adult patients with cirrhosis (102 males, 43 females) from our hospital were enrolled in the study. The only inclusion criterion was the presence of clinical portal hypertension with intrinsic liver cirrhosis. Patients with significant obstructive lung disease, restrictive ventilatory defects, human immunodeficiency virus, severe aortic or mitral stenosis, regurgitation or significant left ventricular dysfunction were excluded. Also excluded were individuals with hepatocellular carcinoma or any other malignancy, known hemostatic disorders other than liver disease, bacterial infection, a clinical history of peripheral venous thrombosis or Budd-Chiari syndrome, spleen resection, lung and liver transplantation and those receiving endoscopic treatment or anticoagulation therapy. A total of 100 patients were considered acceptable and were enrolled in our study. Informed consent was obtained from all patients and the study was carried out according to the principles of the declaration of Helsinki and the guidelines of the institutional ethics committee.

Liver cirrhosis was diagnosed by clinical findings or morphology and liver function of the POPH patients was scored using the Child-Pugh classification (7). Past medical history and social history were recorded. Non-invasive screening for POPH was performed and evaluated by Doppler echocardiography. In order to identify risk factors associated with POPH, the patients were classified into two groups: a POPH group [pulmonary artery systolic pressure (PASP) ≥40 mmHg] and a non-POPH group (PASP <40 mmHg) (8,9).

### Diagnosis of POPH

Pulmonary vascular resistance (PVR) is considered an essential feature of POPH. Diagnostic measurements of PVR are traditionally obtained by right heart catheterization (RHC) (10,11); however, since bleeding complications are a concern among cirrhotic patients, other forms of diagnosis are preferred. Results of Doppler echocardiography are comparable to RHC in non-cirrhotic patients with pulmonary hypertension (12,13). For cirrhotic patients undergoing liver transplantation (14,15), it decreases the requirement for repeated invasive measurements. In our study, PASP (measured by Doppler echocardiography) was used to diagnose POPH.

Noninvasive color Doppler echocardiographies were performed on all patients. The MPAP was calculated from PASP (MPAP = 0.61 PASP + 2 mmHg). PASP values of 38–54 mmHg were considered to represent mild POPH, 55–69 mmHg were moderate and values ≥70 mmHg were severe (16).

### Collection and analysis of blood samples

Blood samples (20 ml) were collected from patients following at least 12 h of fasting. Hemoglobin (Hb) and blood platelet count (BPC) were determined using a Sysmex XE-2100 automated analyzer (Sysmex, Kobe, Japan). Total bilirubin (TBIL) and albumin (ALB) were determined using diazotization and the bromocresol green (BCG) assay method, respectively (Roche Cobas-c702, Germany). D-dimer and high sensitivity C-reactive protein (hs-CRP) levels were detected using the corresponding kits from Sun Biotech Co., Ltd. (Shanghai, China), following the manufacturer’s instructions. Prothrombin time (PT), activated partial prothrombin time (APTT) and fibrinogen (Fib) were determined by routine coagulation methods with a coagulation detector, using a Sysmex CA-6000 automated analyzer (Sysmex, Milton Keynes, UK). Intercellular adhesion molecule 1 (ICAM-1), interferon-α (IFN-α) and tumor necrosis factor-α (TNF-α) were measured by enzyme-linked immunosorbent assay (ELISA).

### Statistical analysis

The SPSS software package for Windows (SPSS version 11.0, SPSS Inc., Chicago, IL, USA) was used for statistical analysis. Continuous data were summarized using mean ± standard deviation (SD) or median (interquartile range), as appropriate. Categorical variables were displayed as frequencies. Statistical analysis was performed using the appropriate parametric or non-parametric tests. Differences between the POPH and non-POPH groups were evaluated by Chi-square test. For continuous data, the assumption of normality was evaluated using a normality test. The Pearson Chi-square test, corrected Chi-square test and Fisher’s exact test were used, as appropriate. Multivariate binary logistic regression was performed to evaluate the correlation between the presence of portal vein thrombosis and thrombotic risk factors with respect to the odds of occurrence for an event. The model was estimated using a backward stepwise method (Wald). In this multivariate analysis, we used a number of variables, including those that were not significant univariate predictors, since they may contribute to a multiple regression model in unforeseen ways due to complex intercorrelations among them. The coefficients obtained from the logistic regression analyses were also expressed in terms of odds ratios (ORs) with 95% confidence intervals. Considering the number of cases, we determined that the final multivariate model should include ≤4 predictors to prevent overfitting. A two-tailed P-value ≤0.05 was considered to indicate a statistically significant difference.

## Results

### Clinical features

Ten of the 100 study patients (10%) were diagnosed with POPH. Five of these (50%) were males aged 30–79 years (bivariate correlation analysis was used) with a mean age of 59.0±24.0 years. Five (50%) were females aged 62–82 years, with a mean age of 73.0±9.0 years. The median age of the 10 POPH patients was 66 years. With regard to the etiology of subjects, eight of the POPH patients had viral cirrhosis [hepatitis B virus (HBV), hepatitis C virus (HCV) or HBV and HCV], one had autoimmune cirrhosis and one had cryptogenic cirrhosis. POPH was not observed in schistosomal or alcoholic cirrhosis patients. Among the ten POPH patients, PASP values were 40–70 mmHg. The severity of POPH in the subjects is listed in Table I.

### Clinical symptoms or signs

The presentations of the decompensate liver cirrhosis and portal hypertension, including jaundice, ascites, splenomegaly, edema and gastrointestinal hemorrhage were observed in the cirrhotic patients with POPH. Although a number of patients with POPH are asymptomatic (17), other POPH patients presented with syndromes and signs of pulmonary hypertension. Two patients presented dyspnea upon exertion, two with syncope, one with chest pain and one with hemoptysis.

### Risk factors analysis

There were no significant differences in age or gender between the POPH and the non-POPH groups (mean age, 66.0±18.0 vs. 61.6±13.9 years; P=0.86, P=0.15, respectively). Of the etiologies studied, only the prevalence of HCV-related cirrhosis patients differed between the two groups (P=0.001). With regards to liver function, we identified that one POPH patient was Child-Pugh grade A, six were grade B and three were grade C. There were no significant differences in Child-Pugh grade between the two groups (P=0.76). With regard to the severity of POPH, liver damage was mild in seven patients, moderate in two patients and severe in one patient. With respect to past medical history only the incidence of portal vein thrombosis differed significantly between the POPH and non-POPH groups (P=0.02; Tables II and III).

The levels of BPC, ALB, TBIL, Hs-CRP, D-dimer, thrombin time (TT), Fib, ICAM-1, IFN-α and TFN-α did not differ significantly between POPH and non-POPH patients. There were no significant differences in the levels of APTT and PT between the two groups. However, the levels of Hb were lower in the POPH group than those in the non-POPH groups (P=0.001). Hb was determined to be a risk factor of POPH by univariate analysis (Table IV).

### Correlation between Child-Pugh class and severity of POPH

**Figure 1.**
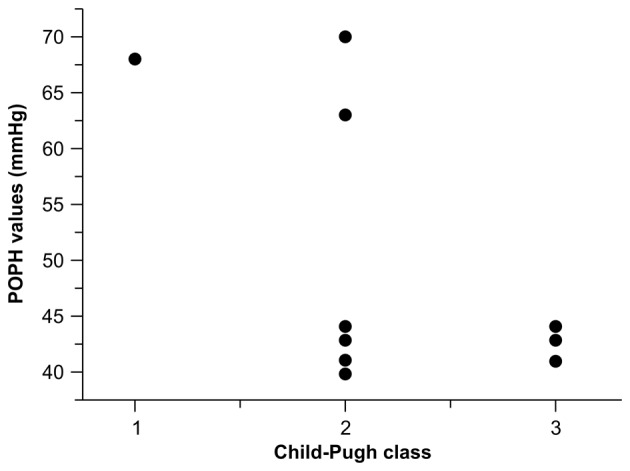
Correlation between Child-Pugh class and POPH values. The x-axis represents the Child-Pugh grades of the POPH patients (1, class A; 2, class B and 3, class C). The y-axis represents the determined POPH values of the POPH patients. Correlation analysis revealed that POPH values were not significantly correlated with Child-Pugh class (R=−0.06, P=0.09). POPH, portopulmonary hypertension.

In our study, a correlation between Child-Pugh class and severity of POPH (reflected by the value of PASP) was investigated. The results revealed that the PASP values were not significantly correlated with Child-Pugh class (R=−0.06, P=0.09; Fig. 1).

### Multivariate analysis

The Hb, HCV, D-dimer and portal vein thrombosis were included in multivariate logistic regression analysis. Only Hb was identified as an independent factor associated with an increased risk of POPH (OR=0.952; Table V).

## Discussion

In the present study, POPH was diagnosed in 10% of patients with cirrhosis. This is within the range of prevalence reported in the literature (20). In previous studies, POPH has been estimated to occur with a prevalence of 16.1% in patients with cirrhosis and refractory ascites and 0.25–4% in patients with cirrhosis without refractory ascites (18,19).

Although our results demonstrated that there are higher percentages of females with cirrhosis that develop POPH, no significant difference was observed in the incidence of POPH across the gender (results not shown). This indicates that males and females with cirrhosis are at risk of developing POPH. These results contradict the findings of a previous study that identified that females are at a higher risk of POPH (11).

In our study, the majority of the POPH patients had viral cirrhosis (HBV and HCV). Viral cirrhosis was the most common type of cirrhosis among our Chinese population; however, our results demonstrated that a higher incidence of POPH exists among patients with viral cirrhosis. While previous research identified that HCV infection was negatively correlated with POPH (11), this was not supported by our data. In contrast, the high incidence of POPH among patients with HCV infection indicates that patients with HCV cirrhosis may be at higher risk of developing POPH.

With regard to the severity of POPH, our results demonstrated that the severity of POPH is unrelated to liver function, as we were unable to identify an association between POPH severity and Child-Pugh class. However, this conclusion is based on a small number of cases and larger studies are required to verify this correlation.

Previously hypothesized risk factors of POPH were not associated with POPH in our study. Age, gender and Child-Pugh class did not differ between the POPH and non-POPH groups. Also, hepatic complications, including GI hemorrhage, hepatorenal syndrome ascites and encephalopathy did not differ between the groups. Similarly, HBP, DM, coronary artery disease, drug use and blood transfusion did not appear to affect the risk of developing POPH and smoking and alcohol use were common in the two groups.

With respect to patient medical histories, only portal vein thrombosis differed between the two groups, indicating that portal vein thrombosis is associated with POPH. These results are supported by a previous study that described microembolism of the lungs in hepatic fibrosis due to recurrent cholangitis. The passage of small emboli to the lungs was attributed to the presence of small hepatic arteriovenous fistulae (21). The correlation between POPH and portal vein thrombosis may be explained by a similar anatomical mechanism. Portal venous obstruction secondary to portal vein thrombosis would likely enlarge such communications, resulting in repeated microembolism of the lungs. This may lead to pulmonary hypertension and, ultimately, congestive failure due to chronic cor pulmonale (21).

Among the laboratory parameters examined in our study, levels of Hb were lower in the POPH group than in the non-POPH group. To our knowledge, a correlation between POPH and Hb has not been described previously. However, it is known that a decrease in Hb leads to a significant increase in cardiac output and exacerbated hyperdynamic splanchnic circulation (22). The hyperdynamic splanchnic circulation is a major contributor to portal hypertension. Therefore, it is not surprising that Hb levels were significantly lower in the POPH group and that Hb level was the only variable independently associated with POPH. These findings indicate that Hb level is an independent risk factor and plays a key role in the development of POPH. While HCV and PVT were also associated with a higher incidence of POPH, these factors were not independent predictors of POPH. Perhaps they contributed to the formation of POPH through interactions with other unknown factors. However, significant differences between the two groups were not observed for any of the other markers that we investigated.

In conclusion, within our study population POPH was present in 10% of cirrhotic patients. It was most common among patients with viral cirrhosis and absent among schistosomal and alcoholic cirrhosis patients. Notably, the severity of POPH was unrelated to liver function (Child-Pugh classification). HCV infection and portal vein thrombosis may play important roles during the development of POPH; however, Hb level is the only significant, independent predictor of POPH. Future studies should examine the mechanistic role of these factors in the development of POPH.

## Figures and Tables

**Table I.** Demographic and clinical features of the ten cirrhotic patients with POPH.

Case	Age (years)	Gender	Etiology	Child-Pugh class	PASP (mmHg)	Degree
1	82	Female	HBV+HCV	A	68	Moderate
2	68	Female	HBV	B	63	Moderate
3	79	Male	HCV	C	44	Mild
4	77	Male	Autoimmune	B	70	Severe
5	82	Female	HBV	C	41	Mild
6	76	Male	HBV	B	44	Mild
7	30	Male	HCV	C	43	Mild
8	62	Female	HBV	B	40	Mild
9	72	Female	Cryptogenic	B	41	Mild
10	36	Male	HBV	B	43	Mild

POPH, portopulmonary hypertension; PASP, pulmonary artery systolic pressure; HBV, hepatitis B virus; HCV, hepatitis C virus.

**Table II.** Comparison of the clinical characteristics between cirrhotic patients with and without POPH.

	POPH	Non-POPH	t/χ^2^	P-value
N	10 (10%)	90 (90%)		
Age (years)	66.0±18.0	61.6±13.9	0.19	0.86
Gender (male/female)	5/5	65/25	2.15	0.15
Etiology				
HBV	5 (50%)	51 (56.7%)	0.10	0.72
HCV	2 (20%)	1 (1.1%)	Fisher’s exact test	0.03
Alcoholic	0 (0%)	5 (5.6%)	Fisher’s exact test	1.00
Schistosomal	0 (0%)	7 (7.8%)	0.07	0.79
Autoimmune	1 (10%)	8 (8.9%)	0.01	0.91
Cryptogenic	1 (10%)	18 (20%)	0.59	0.44
HBV+HCV	1 (10%)	0 (0%)	Fisher’s exact test	0.10
Child-Pugh class				
A	1	13		
B	6	56	0.55	0.76
C	3	21		
Severity of POPH				
Mild	7	0		
Moderate	2	0		
Severe	1	0		

Age is expressed as mean ± standard deviation (SD) and categorical variables are displayed as frequencies (%). POPH, portopulmonary hypertension; HBV, hepatitis B virus; HCV, hepatitis C virus.

**Table III.** Comparison of medical histories between patients with and without POPH.

Variable	POPH (%)	Non-POPH (%)	χ^2^	P-value
N	10	90		
GI hemorrhage	50	27.8	2.12	0.15
Hepatic encephalopathy	20	23.3	0.06	0.81
Hepatorenal syndrome	20	21.1	0.01	0.94
Ascites	80	77.8	0.03	0.87
Smoking	20	23.3	0.06	0.81
Alcohol abuse	10	10.0	0.00	1.00
High blood pressure	20	26.7	0.21	0.65
Diabetes mellitus	10	11.1	0.01	0.92
Coronary artery disease	10	5.6	0.32	0.58
Blood transfusion	60	51.1	0.29	0.59
Portal vein thromosis	50	16.7	5.63	0.04
Drug use	10	0.0	Fisher’s exact test	0.10^a^

POPH, portopulmonary hypertension; GI, gastrointestinal.

a Fisher’s exact test was conducted since the data was not suitable for Chi-square test.

**Table IV.** Comparison of laboratory results between patients with and without POPH.

Variable	POPH	Non-POPH	t	P-value
Hb (g/l)	72.6±14.2	98.6±24.1	2.46	0.00
BPC (×10^9^/l)	82.1±44.3	83.4±46.9	−0.08	0.93
TBIL (μmol/l)	30.1±12.3	40.8±25.4	1.38	0.17
ALB (g/l)	26.1±5.5	26.8±6.1	0.33	0.75
Hs-CRP (mg/l)	9.8±9.0	17.1±2.4	0.93	0.36
D-dimer (mg/l)	0.3±0.2	0.6±0.1	1.09	0.28
APTT (sec)	39.3±9.2	36.6±10.4	−0.79	0.43
PT (sec)	14.7±2.8	14.1±2.9	−0.61	0.54
TT (sec)	18.7±2.2	21.2±5.1	1.45	0.14
Fibrinogen (g/l)	2.4±0.7	2.8±0.3	0.40	0.69
ICAM-1(ng/ml)	14.9±10.1	19.3±2.5	0.54	0.59
IFN-α (pg/ml)	23.0±13.7	37.4±16.6	0.68	0.50
TNF-α (pg/ml)	14.5±4.7	21.3±3.2	0.66	0.51

Data are presented as mean ± standard deviation (SD). POPH, portopulmonary hypertension; Hb, hemoglobin; BPC, blood platelet count; TBIL, total bilirubin; ALB, albumin; hs-CRP, high sensitivity C-reactive protein; APTT, activated partial prothrombin time; PT, prothrombin time; TT, thrombin time; ICAM-1, intercellular adhesion molecule 1; IFN, interferon; TNF, tumor necrosis factor.

**Table V.** Results of backward, stepwise logistic regression analysis of the correlation between POPH and Hb level, HCV, D-dimer level and portal vein thrombosis.

Variables	β-value	SE	Wald value	P-value	OR	95% CI
Hb	−0.049	0.021	5.478	0.02	0.952	0.913–0.992
HCV	2.464	1.787	1.901	0.17	11.746	0.354–389.915
D-D	−0.020	0.025	0.625	0.43	0.981	0.934–1.0290
PVT	1.129	0.778	2.296	0.13	3.252	0.707–14.949

POPH, portopulmonary hypertension; SE, standard error; OR, odds ratio; CI, confidence interval; Hb, hemoglobin; D-D, D-dimer; PVT, portal vein thrombosis.
